# Association of Dietary Patterns, Suspected Sarcopenia, and Frailty Syndrome among Older Adults in Poland—A Cross-Sectional Study

**DOI:** 10.3390/nu16183090

**Published:** 2024-09-13

**Authors:** Robert Gajda, Marzena Jeżewska-Zychowicz, Ewa Raczkowska, Karolina Rak, Małgorzata Szymala-Pędzik, Łukasz Noculak, Małgorzata Sobieszczańska

**Affiliations:** 1 Department of Human Nutrition, Faculty of Biotechnology and Food Sciences, Wrocław University of Environmental and Life Science, Norwida 25, 50-375 Wroclaw, Poland; ewa.raczkowska@upwr.edu.pl (E.R.);; 2 Department of Food Market and Consumer Research, Institute of Human Nutrition Sciences, Warsaw University of Life Sciences (SGGW-WULS), Nowoursynowska 159C, 02-776 Warsaw, Poland; marzena_jezewska_zychowicz@sggw.edu.pl; 3 Clinic Department of Geriatrics and Internal Diseases, Faculty of Medicine, Wroclaw Medical University, M. Curie-Skłodowskiej 66, 50-369 Wroclaw, Polandmalgorzata.sobieszczanska@umw.edu.pl (M.S.)

**Keywords:** dietary patterns, sarcopenia, frailty syndrome, older adults

## Abstract

Background: The association of sarcopenia and frailty syndrome with dietary patterns is not yet well recognized. The aim: The aim of the study was to evaluate the association among dietary patterns, suspected sarcopenia, and frailty syndrome among older people in Poland. Methods: The study was conducted in 2022 and 2023 among people aged 55 and older. The sample was chosen arbitrarily. The following questionnaires were used in the study: the KomPAN (assessment of frequency of food intake and sociodemographic characteristics), the SARC-F (assessment of risk of sarcopenia), and the EFS (diagnosis of frailty syndrome). To confirm the suspicion of sarcopenia, muscle strength was assessed using the HGS and FTSST, and physical fitness was assessed using the GST. Based on the frequency of food consumption, 11 DPs (factors) were selected using PCA analysis. SARC-F, HGS, FTSST, and GST results were used to identify homogeneous groups (clusters) using cluster analysis, a k-means method. Results: Two clusters were identified: cluster 1 (the non-sarcopenic cluster, or nSC) and cluster 2 (the sarcopenic cluster, or SC). Associations between variables were assessed using logistic regression. Suspected sarcopenia was found in 32.0% of respondents, more in men than women, and more among those either over 75 or 65 and under. EFS results showed that the risk (22.1%) or presence of frailty syndrome (23.8%) was more common in men than women and more common in those aged 75 and older than in other age groups. Male gender; older age; and unfavorable dietary patterns, i.e., consumption of white bread and bakery products, white rice and pasta, butter, and potatoes (factor 1) and cheese, cured meat, smoked sausages, and hot dogs (factor 9), increased the likelihood of sarcopenia and frailty syndrome, while the pattern associated with fruit and water consumption (factor 7) had the opposite effect. Conclusions: Confirmation of the importance of dietary patterns in the etiology and pathogenesis of sarcopenia and frailty syndrome should be documented in prospective cohort studies.

## 1. Introduction

An improvement in living conditions and advances in healthcare cause a prolongation of lifespan, resulting successively in a global ageing process. The European population aged 65 years and older in 2020 constituted 21%, in contrast to 16% in 2001 year [1,2]. In Poland, according to the latest demographic data, the subpopulation of older people aged 60 and over in 2023 accounted for 26.3% of the total population, and the proportion has been growing steadily year over year since 2006 [3].

Frailty syndrome is one of the specific so-called giant geriatric syndromes. The operational definition of frailty syndrome dates back to 2001, when Linda Fried et al. proposed a phenotype fulfilling at least three of the following five criteria: unintentional weight loss (4.5 kg in the past year), self-reported exhaustion, weakness (measured with hand grip strength), slow walking speed, and physical inactivity [4]. At present, there is still no consensus on a comprehensive definition of the term “frailty”. Generally, it is a dynamic, reversible, multifactorial clinical status linked to a loss of physiological reserve and a failure of homeostasis, expressed as enhanced vulnerability to exposure to exogenous or endogenous stressors, which implies various negative healthcare conditions [5,6,7].

Frailty syndrome shows a prevalence ranging from 12% to 26% and the pre-frail state up to 45% in those aged over 60 years using different frailty measures. A problem arises because different tools yield variable results concerning frailty severity, and the predictive accuracy of the tests is often questionable. Therefore, there is no assessment scale that serves as the gold standard in clinical practice. This fact implies the need for standardization and accurate guidelines concerning the diagnosis of frailty syndrome in older adults [8,9,10,11]. Nevertheless, the latest large population-based study of the health status and quality of life of older adults in Poland, the results of which are included in the 2021 PolSenior 2 Report [12], found that frailty syndrome affected 15.9% of those surveyed. The syndrome was identified more often among older women (17.4%) than among older men (13.8%). With age, the prevalence of frailty syndrome increased significantly beyond the age of 70, from 11.6% in those aged 70–74 to 70.8% among 90-year-olds [13].

Frailty syndrome frequently, although not always, coexists with sarcopenia—an age-dependent muscle failure occurring with a loss of muscle mass and consequently reduced muscle strength that is considered a key characteristic of sarcopenia, playing, according to the updated EWGSOP2 (European Working Group on Sarcopenia in Older People 2) recommendations, a predominant role in the physical performance and functional state of older people [14,15]. Landi et al. [16] even proposed that sarcopenia could be treated as the biological substrate of frailty. Similar to frailty syndrome, sarcopenia is a major geriatric problem. The diagnosis of sarcopenia is carried out in four steps. It begins with screening using the SARC-F questionnaire. Those at risk are then assessed for muscle strength using hand grip strength (HGS) or the “five times sit-to-stand test” (FTSST). If low values are obtained on these tests, sarcopenia is suspected. In clinical practice, to confirm sarcopenia, muscle strength assessment should be confirmed by muscle volume assessment (BIA, DEXA, CT, or MRI), although the suspicion of sarcopenia is sufficient to implement treatment. The fourth step in the diagnosis of sarcopenia is to assess its severity. For this purpose, the results of physical fitness tests are analyzed, including the gait speed test (GST), “get up and go” test, short physical performance battery test, and 400 m walk test [15].

The prevalence of sarcopenia in the general world population is estimated at 8–13% among women and 8–12% among men. It is believed to more often affect non-Asian populations [17]. Among people over 65 years of age receiving long-term care, the prevalence ranges from 17.7% to 73.3%; such significant discrepancies are due to differences in the methodology adopted by the researchers [18]. In the 2021 PolSenior 2 report [12], it was indicated that HGS indicative of suspected sarcopenia [15] affected 11.3% of people aged 60 and older, comparably between men and women, displaying a significant increase with age [19]. FTSST indicative of suspected sarcopenia [15] affected an average of 42.0% of older subjects, with a higher rate in women (47.6%) than men (34.3%), and it increased significantly with age [19].

Since the mid-1990s, a holistic approach to dietary assessment, which involves evaluating the impact of dietary patterns (DPs) on health, has gained prominence, while the traditional approach has begun to see many limitations [20,21]. It is believed that traditional nutritional research, based on the study of the assessment of individual food components (a nutrient-specific approach to health), may underestimate the overall impact of food on health, cause misinterpretation of the results, and, as a result, be responsible for the formulation of erroneous dietary recommendations [22]. The following are pointed out as limitations of traditional research: the complex composition of food, the fact that food and nutrients are consumed in different combinations, the possible occurrence of interactions between food components and their potential cumulative or disruptive effect on the multifactorial etiology of diseases, the interrelationships between causal factors, and the influence of dietary habits on the metabolism of a nutrient in the body [21,22,23,24,25,26,27].

The first definition of dietary patterns was given by Frank Hu [21], stating that “dietary patterns represent a set of a number of characteristic jointly occurring features describing human nutrition.” The current thinking is that dietary patterns are a complex construct involving many features of nutrition, including the amount and combinations of foods that are habitually consumed, nutrients and food products, and interactions between dietary components [21,22,24,27]. The number of studies and published papers that have identified dietary patterns and assessed their impact on health is steadily increasing [26,27].

There is a known assessment among the incidence of sarcopenia, energy intake, protein intake [28,29,30,31] (including the consumption of branched-chain amino acids and leucine [29,30]), and vitamin D3 intake [31,32]. The association between frailty syndrome and nutritional deficiencies was also recognized [33], including deficiencies of vitamin D_3_, omega-3 fatty acids [34], and magnesium [35]. In contrast, to the best of the knowledge of the authors of this paper, the world literature does not recognize numerous studies on the association between dietary patterns, sarcopenia, and frailty syndrome. It was hypothesized that the occurrence of sarcopenia and frailty syndrome is related to unfavorable dietary patterns. In contrast, favorable dietary patterns were hypothesized to play a protective role. In this context, the aim of the study was to assess the association of dietary patterns, suspected sarcopenia, and frailty syndrome among older people in Poland.

## 2. Materials and Methods

### 2.1. Study Design and Sample

This cross-sectional study was conducted between August 2022 to December 2023 with participants aged 55 and older. The sample was chosen arbitrarily. A long-term care institution in Wroclaw, housing a Social Welfare Home and a Day Care Center, was asked to help recruit participants for the study; with the assistance of the Wroclaw Center for Social Development in Wroclaw, the study was conducted in two Senior Citizens’ Clubs. In addition, students from the Faculty of Medicine at Wroclaw Medical University helped recruit and survey older people living in the community. A total of 172 direct interviews were conducted at the Social Welfare Home (39), Day Care Center (10), and Senior Citizens’ Clubs (64) and among community-dwelling seniors—alone or with family (59).

The study was conducted in accordance with the Declaration of Helsinki [36]. Participation in the study was voluntary. Informed consent to participate in the study, permission to publish the results of the study, and permission to process personal data for scientific purposes were obtained from all participants. The study was approved by the Bioethics Committee at the Wroclaw Medical University, opinion number KB-551/2022.

### 2.2. Questionnaire

In a direct interview, questions from the KomPAN questionnaire [37] were used to assess the frequency of consumption of 32 food groups, as well as socio-demographic characteristics (gender, age, place of residence, and education). To determine the frequency of food intake, people were asked about the consumption of different groups of cereal products (4 items), dairy products (4 items), meat products and dishes (3 items), fats (3 items), egg dishes and snacks (1 item), fish dishes and snacks (1 item), pulses (1 item), potato dishes (not including chips and crisps) (1 item), fruit (1 item), vegetables (1 item), fried foods (meat and flour) (1 item), sweets (1 item), fast food (1 item), concentrates and ready-made soups (1 item), various canned foods (2 items), various juices (2 items), various drinks (3 items), and water (1 item). The participants reported the habitual frequency of eating each food during the 12 months preceding the survey using one of the following answers: 1—less than once a month or never; 2—1–3 times a month; 3—once a week; 4—a few times a week; 5—once a day; or 6—a few times a day [37]. During the data analysis, the answers were converted to reflect the daily frequency of intake, ranging from 0—less than once a month or never; 0.06—1–3 times a month; 0.14—once a week; 0.5—a few times a week; 1—once a day; and 2—a few times a day [38].

In the present study, screening for sarcopenia risk was performed using the SARC-F questionnaire. The maximum total score on this questionnaire was 10. The risk of sarcopenia was identified by a score of 4 points or more [39]. In addition, measurements assessing muscle strength were taken to confirm suspected sarcopenia: hand grip strength (HGS) [40], the “five times sit-to-stand test” (FTSST) [41], and measurement to assess physical fitness through the gait speed test (GST) [15]. According to the current EWGSOP2 recommendations, a person can be suspected to have sarcopenia when demonstrating a low gait speed (≤0.8 m/s) and grip strength below the cut-off points (i.e., female < 16 kg; men < 27 kg) [15] and when the “five times sit-to-stand test” takes longer than 15 s (>15 s), which indicates a weakening of thigh muscle strength [41].

Functional measurements carried out to confirm suspected sarcopenia were performed according to the following procedures:

1. HGS. A standard hydraulic hand dynamometer with 90 kg capacity (12-0240; Fabricati Enterprises, Inc.) was used in our measurements. The subjects were asked to sit comfortably with their knees, pelvis, and back positioned at approximately 90 degrees. The elbow of the dominant subject’s hand was flexed to a 90-degree angle, the forearm was neutral, the wrist was kept between 0 and 15 degrees of ulnar deviation, and the shoulder was abducted and neutrally rotated. The dynamometer was positioned vertically and in line with the forearm. The subject was allowed one trial before the actual measurement. The hand grip strength was recorded when the subject was instructed to squeeze the dynamometer’s handle with maximal strength. The HGS value was the average of the three trials, with a 15 s intertrial rest interval between each trial [42].
2. FTSST. This test consists of standing independently from a chair five times as quickly as possible without pushing off the chair. The subject kept both arms crossed in front of the chest and sat comfortably with the back supported by the back of the chair, and the knees were required to be maximally extended when standing from the chair. The person was given instructions about the task prior to the test and had two opportunities to practice. A stopwatch was used to record the performance in seconds from the starting seated position to the terminal seated position [43].
3. GST. Gait speed was measured over a 4 m flat course at the subject’s usual pace and is expressed in m/s. The subject was positioned at the starting point in a standing, relaxed position and, at the start signal, was asked to walk a fixed distance [44].

Frailty syndrome (FS) risk screening was conducted using the Edmonton Frail Scale (EFS). The maximum total score using this scale was 17. The absence of frailty syndrome was identified in the range of 0–4 points; scoring 5–6 points indicated a risk of frailty syndrome, 7–8 points a mild degree, 9–10 points a moderate degree, and 11–17 points a severe degree of frailty syndrome [45].

Physicians from the Department of Geriatrics at Wroclaw Medical University carried out the above functional tests and measurements.

### 2.3. Statistical Analysis

Descriptive statistics were used for quantitative variables, and frequency analysis for qualitative variables.

A factor analysis was conducted to derive dietary patterns, or DP (factors), based on the frequency of consumption of 24 food groups. An orthogonal (Varimax) transformation rotated the factors. The number of factors was based on the following criteria: components with an eigenvalue of 0.1, a scree plot test, and the interpretability of the factors. Food items were considered to load on a factor if they had an absolute correlation of 0.5 with it. DPs (factors) were derived by principal component analyses (PCAs) to which variables describing the daily frequency of eating some foods were introduced. The factorability of data was confirmed when the Kaiser–Meyer–Olkin (KMO) measure of sampling adequacy and Bartlett’s test of sphericity showed statistical significance. Buckwheat, oats, wholegrain pasta or other coarse-ground groats; fried foods (e.g., meat or flour-based foods such as dumplings, pancakes, etc.); vegetable oils or margarines or mixes of butter and margarines; milk (including flavored milk, hot chocolate, and lattes); white meat, e.g., chicken, turkey, and rabbit; vegetables; and sweets, e.g., confectionary, biscuits, cakes, chocolate bars, cereal bars, were not included in the identified factors due to correlations coefficients lower than 0.5.

Based on the results of the SARC-F, HGS, FTSST, and GST, segmentation was carried out to distinguish homogeneous groups according to indicators related to sarcopenia using the non-hierarchical k-means method. Two clusters were identified, namely, cluster 1, or the non-sarcopenic cluster (nSC, 117 people) and cluster 2, or the sarcopenic cluster (SC, 55 people). The clusters differed significantly in the score for each variable, with the exception of the score for the muscle strength test. The correctness of the cluster separation was assessed using the CCC (cubic clustering criterion) statistic and the pseudo-F statistic.

Six logistic regression models with a dichotomous dependent variable were used. Logistic regression analysis was used to verify associations between gender; age; identified DP; and such dependent variables as SARC-F—model 1, GST—model 2, FTSST—model 3, HGS—model 4, and EFS—model 5. Model 6 showed adherence to the dependent variable called sarcopenic cluster (SC) and explanatory variables such as gender, age, DP, and incidence of FS. Odds ratios (ORs) represented the probability of adherence to the group characterized by sarcopenia and frailty syndrome symptoms. The reference groups (OR = 1.00) were those not representing these symptoms and those defined by female gender, age 65 and under, and no DP affiliation. Wald’s test was used to assess the significance of ORs. Only statistically significant explanatory variables were used in the model created. The Hosmer and Lemeshow goodness-of-fit test, the explained variation of survival (Nagelkerke R^2^), and the correctness of predictions were used to assess the quality of the resulting model.

The statistical analysis was performed using IBM SPSS Statistics for Windows, version 29.0 (IBM Corp, Armonk, NY, USA).

## 3. Results

The study group comprised 172 people aged 55 and over, 79.1% of whom were women. The average age was 72.9 years (SD ± 7.67). The largest share of the study group was made up of people with vocational education (47.7%); living in cities with more than 100,000 residents (76.1%); and living in their own home/apartment, including alone (39.5%) or with a partner (24.4%). Detailed characteristics of the study group are shown in Table 1.

The factor loading matrix for eleven dietary patterns (factors) identified by principal component analysis is presented in Table 2. The KMO value was 0.684. Bartlett’s test gave a significance of *p* < 0.0001. They explained 61.8% of the total variance. Characteristics of the dietary patterns are shown in Table 3. The average multiplicity of product consumption in the extracted dietary patterns was highest for factor 7 (fruit and water) and factor 1 (white bread and bakery products, white rice and pasta, butter, and potatoes). The lowest average multiplicity of product consumption was recorded for factors 11 (fast food) and 6 (red meat, tinned meats, and energy drinks).

Characteristics of the study group, including indicators of sarcopenia and frailty syndrome as well as the gender and age of the subjects, are shown in Table 4.

A risk of sarcopenia (SARC-F ≥ 4 points) was found in 32.0% of respondents, with more men (63.9%) than women (23.5%) having scores confirming the risk of sarcopenia. Two functional tests confirmed the risk of sarcopenia in more men than women, i.e., the HGS (38.9% vs. 10.3%) and FTSST (52.8% vs. 25.0%). The GST confirmed the risk of sarcopenia in more women than men. The risk of sarcopenia was highest among respondents aged 75 and older (46.6%) but was also high among those aged 65 and younger (40.7%). Suspected sarcopenia was confirmed by the FTSST in the same age groups (44.8% and 29.6%, respectively). The GST showed a higher percentage of suspected sarcopenia in the respondents aged 66–75. The HGS test did not confirm the differences in suspected sarcopenia by age. The EFS indicated the risk (22.1%) or presence of frailty syndrome (23.8%) in 45.9% of respondents. More men (63.9%) than women (41.2%) and more people aged 75 and over (63.8%) and aged 71–75 (50.0%) than in younger age groups were characterized by the risk or presence of frailty syndrome (Table 4).

Logistic regression results showing the predictive effects of gender, age, and dietary patterns on the incidence of suspected sarcopenia and frailty syndrome are shown in Table 5. The models (1–5) were statistically significant when compared to the null model (*p* < 0.001), explaining 37.6%, 45.4%, 26.7%, 34.8%, and 41.% of the variation of survival (Nagelkerke R2), respectively, and correctly predicted 75.6%, 79.1%, 76.2%, 87.2%, and 75.0% of cases, respectively. The Hosmer and Lemeshow goodness-of-fit test for the models yielded the following results: 0.449, 0.340, 0.432, 0.594, and 0.928, respectively. Men were more likely than women to have a SARC-F score ≥ 4 (over five times greater), an FTSST score > 15 s (over three times greater), an HGS scores indicative of handgrip strength (over nine times greater), and a GST score ≤ 0.8 m/s (over three times greater). Each additional year of age (over 65) increased the risk score for sarcopenia by 7%, reduced muscle strength according to the FTSST index by 6%, and increased the risk or presence of frailty syndrome by 8%.

In people who consumed white bread and bakery products, white rice and pasta, butter, and potatoes (factor 1) and cheese, cured meat, and smoked sausages (factor 9), there was a significantly increased chance of suspected sarcopenia based on gait speed assessment (Model 2), while more frequent consumption of fruit and water (factor 7) significantly reduced the chances of this indicator occurring. The occurrence of pre-frail or frail condition (Model 5) was more likely with consumption of white bread and bakery products, white rice and pasta, butter, and potatoes (factor 1) and with less frequent consumption of fruit and water (factor 7). The other dietary patterns showed no predictive effect on the dependent variables. Detailed characteristics of models of SARC-F, GST, FTSST, HGS, and EFS results in relation to gender, age, and all analyzed dietary patterns (factors) in the study group are presented in the Appendix A (Table A1, Table A2, Table A3, Table A4 and Table A5).

Logistic regression results showing the predictive effect of age, dietary patterns, and frailty syndrome on the incidence of suspected sarcopenia (SC affiliation)—Model 6 are shown in Table 6. The model was statistically significant when compared to the null model (*p* < 0.001), explaining 54.7% of the variation of survival (Nagelkerke R2) and correctly predicted 80.8% of cases. The Hosmer and Lemeshow goodness of fit for the model was 0.929. Men were more than five times as likely as women to belong to SC. The presence of pre-frail or frail condition increased the probability of belonging to SC by more than 16 times. People who consumed white bread and bakery products, white rice and pasta, butter, and potatoes (factor 1) were almost 13 times more likely to have sarcopenia than those who did not consume these products. People who consumed cheese, cured meat, and smoked sausages (factor 9) were almost 36 times more likely to have sarcopenia than those who did not consume these products.

## 4. Discussion

Diagnosis of frailty syndrome is difficult due to its multifactorial nature [5,6,7] and a lack of standardized diagnostic criteria and standardized measurement tools [8,9,10,11]. It is much easier to diagnose sarcopenia. This is due to the simple and updated definition of this condition [15,16,18] and the relatively well-standardized diagnostic criteria [15]. A systematic increase in the number of completed studies, including meta-analyses, in the last 10 years provides increasing evidence of the association of sarcopenia and frailty syndrome with nutritional deficiencies, especially dietary deficiencies in energy, protein, branched-chain amino acids, vitamin D, antioxidant vitamins, and many other biologically active substances [28,29,30,31,32,33,46]. It is now believed that traditional nutritional research, based on the study of the association of individual food components (nutrient-specific approach) with health, may underestimate the overall impact of food on health, cause misinterpretation of the results, and, as a result, be responsible for the formulation of erroneous dietary recommendations [22]. One of the more common holistic ways of assessing the impact of the whole diet on health is through dietary pattern analysis [20,21]. In the context of the problem posed in this way and the scarcity of data, the authors evaluated the association of dietary patterns, sarcopenia, and frailty syndrome among older people in Poland.

Available global epidemiological data indicate that the prevalence of frailty syndrome ranges from 12–26% of people over the age of 60. Considering only the risk assessment of frailty syndrome, this percentage rises to 45%. As already mentioned, the data discrepancy problem is caused by the use of different measurement scales [8,9,10,11]. The most recent population-based study of the health status and quality of life of older people in Poland, PolSenior 2, indicated the presence of frailty syndrome in 15.9% of respondents [13]. In our study using the Edmonton Frail Scale (EFS) [45], frailty syndrome was identified in 23.8% or its risk in 22.1% of respondents.

The prevalence of sarcopenia in the general world population is estimated at 8–13% among both men and women [17]. In the PolSenior 2 report, functional tests indicative of suspected sarcopenia indicated its presence in 11.3% (HGS test) or even 42.0% (FTSST) of respondents [19]. A meta-analysis including 1686 participants aged 69 years with weak grip strength and/or slowed FTSST, indicating probable sarcopenia, revealed that the SARC-F showed suspected sarcopenia in only 19% of the examined patients [47]. In this study, using the SARC-F questionnaire [39], suspected sarcopenia was found in 32.0% of respondents. This relatively high percentage of people at risk for sarcopenia can be explained in two ways. First, this is due to the sampling method. In our survey, a significant percentage of respondents were residents of long-term care institutions (22.7% of all respondents). Among the older people receiving such care, the prevalence of sarcopenia increases and ranges from 17.7% to 73.3%, with such significant discrepancies due to differences in the methodology adopted by the researchers [18]. Secondly, the prevalence of sarcopenia in the elderly population is increasing over time, and these newer research findings may show a higher rate.

While the results of our own study and global studies on the prevalence of sarcopenia and frailty syndrome do not show large discrepancies, the differences by gender and age are puzzling. Typically, the incidence of sarcopenia and frailty syndrome is higher among women than men and increases with age [13,19]. Although, in our study, logistic regression models showed that the risk of sarcopenia (a 7% increase with each additional year after age 65) and the incidence of frailty syndrome (an 8% increase) increase with age, sarcopenia was also frequently identified in those aged 65 or younger. This situation can be explained by the significant percentage of residents of long-term care institutions aged 65 and under participating in the study (23.1% of all residents) in relation to the total percentage of participants in the other study groups of the same age (14.6%). As we already know, residents of long-term care institutions are more likely to experience sarcopenia because of a lack of physical activity [18]. Most questionable is the result reporting that more men than women experienced sarcopenia and frailty syndrome. Again, men were more likely to be surveyed in a long-term care institution (51.3% of all residents in that institution) than in the other study groups (a total of 13.0% of all respondents in those other groups), which may explain this result.

The diagnosis of sarcopenia begins with a risk assessment using the SARC-F questionnaire and then confirms this risk with indicators of muscle strength (muscle quality) using the HGS and FTSST [15]. Our study showed that more men than women and more people in two specific age groups (>75 years and ≤ 65 years) than in the other age groups were at risk for sarcopenia, according to the SARC-F questionnaire. In these groups, more people had FTSST scores indicative of suspected sarcopenia. The HGS test confirmed such an association in the case of gender. No such association was confirmed for the GST. According to the diagnostic procedure for sarcopenia, the GST is used to assess the severity of the disease (the fourth stage of diagnosis) [15] and is a fitness test. Thus, it seems that the GST does not necessarily confirm the severity of the disease and, at the same time, may indicate low physical fitness among those not experiencing sarcopenia risk, which may not confirm a direct correlation with sarcopenia risk. Nevertheless, the logistic regression model confirmed a threefold higher chance of fitness decline according to the GST index among men than women. In addition, a large population-based Polish study among people aged 60 and older found that slow gait speed predominated among the frailty criteria in older Poles (56.3%), which suggests that this component would be useful in tailored protective and therapeutic actions to prevent frailty syndrome. The less frequent frailty criteria were, in order, subjective weakness (26.9%), exhaustion (19.2%), low physical activity (16.5%), and body mass loss (9.4%) [48].

The strong association of frailty syndrome with sarcopenia is reported by studies [16,49]. In our study, the presence of pre-frail or frail condition increased the likelihood of belonging to the sarcopenia cluster by more than 16 times. It was documented that sarcopenia has a modulating role in the clinical course of frailty. It was revealed in 1538 elderly participants (74.73 ± 5.73; 45.5% men) that transitions from robustness to pre-frailty and frailty were more frequent in sarcopenic than in non-sarcopenic persons. Sarcopenia was associated with an increased risk of progression from robustness to pre-frailty, from pre-frailty to frailty, and from non-frailty to frailty [49]. Both frailty syndrome and sarcopenia, if untreated, can put older persons at higher risk of falls and fractures, increased morbidity, prolonged hospitalizations, disability, need for institutionalization, and, finally, the threat of fatal outcomes [11,50].

It is known that physical activity and a balanced diet, especially including high-protein products and vitamin D3 supplementation, are significant contributors to the prevention of sarcopenia and frailty and to restoration from these conditions, improving the quality of life of older people [31,46,51,52]. Protective effects, particularly against sarcopenia, have been shown with dietary recommendations related to the consumption of optimal amounts of dietary energy and protein [28,29,30,31,46]; supplementation with branched-chain amino acids, including leucine [29,30]; and vitamin D [31,32]. The importance of a number of biologically active compounds in reducing frailty syndrome has been evaluated, including antioxidants [46,53], vitamin D3, omega-3 fatty acids [34], and magnesium [35]. The association between frailty syndrome and abnormal diet quality was also recognized [33,46,54,55,56]. The association of dietary patterns discovered by the “a posteriori” (data-driven) method [22,24] with these conditions, as well as the importance of dietary patterns discovered by this method in the etiology and pathogenesis of sarcopenia and frailty syndrome, has rarely been identified. The effect of the whole diet on the reduction of sarcopenia and frailty syndrome can only be assessed by referring to data based on indicators of diet quality, that is, predefined dietary patterns selected “a priori” (based on available knowledge) [22,24].

Using the Mediterranean Diet Score (MDS) to assess a priori predefined dietary patterns showed that older people living in communities with healthier diets showed a significantly lower risk of frailty, including sarcopenia [54,57]. Considering the individual criteria of frailty, there was a significant association among weight loss, low physical activity, a low GST, and a low MDS [54]. A similar association was shown in studies using two other dietary indicators, i.e., the Alternative Healthy Eating Index (aHEI) and Dietary Approaches to Stop Hypertension (DASH) [57]. In their study, Chan et al. [56] did not confirm any association between MDS and frailty syndrome but confirmed such an association using another diet quality index: the Diet Quality Index–International (DQI-I). This study found that those with higher DQI-I scores (which represent a balanced diet regarding energy and nutrient intake) had a reduced risk of frailty, even after differentiating by gender and age [56]. Finally, a longitudinal study analysis using the Diet Quality Index–Revised (DQI-R) found that the DQI-R score in a cohort of older men was inversely associated with frailty status during two measurements taken 4.6 years apart on average [55].

In our study, in which a posteriori dietary patterns were identified using a database of the intake of 32 food groups, an association was found between unfavorable dietary patterns and the incidence of sarcopenia and frailty syndrome. An inverse association between a favorable dietary pattern and GST index values indicative of sarcopenia was also demonstrated.

Among those more likely to consume white bread and bakery products, white rice and pasta, butter, and potatoes (Factor 1), there was a significantly increased chance of sarcopenia based on GST score (Model 2) and pre-frailty and frailty (Model 5). At the same time, logistic regression analysis confirmed a 13-fold greater chance of sarcopenia among those who consumed these products. A study by Shikany et al. [55] found that a higher intake of carbohydrates and dietary fiber protected against frailty syndrome, while a higher intake of fats showed the opposite effect. Perhaps the effect of the dietary pattern we identified (Factor 1) in stimulating sarcopenia and frailty syndrome occurs because the effect of fat intake (butter) and lower dietary fiber intake (potatoes and refined grain products) is stronger than the protective contribution of carbohydrates in this pattern. In contrast, a study by Koimoto et al. [35] found that older people with a risk of frailty syndrome consumed slightly more dietary carbohydrates than those without the risk.

Most studies report that higher protein intake is associated with a lower risk of sarcopenia and frailty syndrome [28,29,30,31]. It has also been shown that the source of protein does not matter in differentiating the incidence of these conditions (both animal sources, i.e., fish and shellfish, meat, eggs, and dairy products, and plant sources, i.e., cereals, potatoes, fruits, and vegetables) [58]. Although amino acid intake was inversely associated with sarcopenia and frailty syndrome, the association of total protein intake was stronger than that of any single amino acid [58]. However, it is also often said that whey proteins, including leucine, are particularly important in reducing the risk of sarcopenia [29,30]. However, our study showed that the source of protein matters. It was found that those who consumed cheese, cured meat, smoked sausages, and hot dogs (factor 9) more frequently had a significantly increased chance of sarcopenia based on GST index assessment (Model 2) and the occurrence of pre-frailty and frailty (Model 5). At the same time, logistic regression analysis confirmed as much as a 36-fold higher chance of sarcopenia among those who consumed these products in relation to those who did not consume such foods. This situation can be explained by the high intake, in this dietary pattern, not only of protein but also of fat. As we know, fat intake is directly proportionally correlated with frailty syndrome [55]. Other potential factors in processed protein products that may contribute to sarcopenia and frailty syndrome need to be confirmed in studies.

More frequent consumption of fruit and water (factor 7) significantly reduced the chance of the GST index (Model 2) indicating sarcopenia. Studies indicate that antioxidants [46,53]; micronutrients such as carotene, cryptoxanthin, alpha-tocopherol, vitamin B6, folate, and vitamin C [57]; and dietary fiber [55], of which fruits are a source, have protective effects in the etiology of sarcopenia and frailty syndrome. It is likely that these factors in this dietary pattern (factor 7) had a major protective function in the etiology of these diseases. However, there have been studies showing that fruit consumption was not associated with a reduction in frailty syndrome [56].

To the best of our knowledge, this study on the association of a posteriori emergent dietary patterns with sarcopenia and frailty syndrome is one of the few in the literature. However, it has several limitations. First of all, the study is cross-sectional; therefore, the significance of the dietary patterns identified in the study in the etiology of sarcopenia and frailty syndrome cannot be assessed due to the unknown direction of influence and cause-and-effect association. In addition, the non-probabilistic sampling method did not allow a representative sample to emerge, which limits the ability to conclusions from the results obtained. Furthermore, the overrepresentation of male respondents and young adults in a long-term care institution, where one would expect a higher percentage of people with sarcopenia and frailty syndrome, may have influenced the association of these conditions with gender and age in unexpected ways.

## 5. Conclusions

Frailty syndrome and sarcopenia seem to share common pathophysiological markers that involve cellular aging and endocrine metabolism regulation pathways, including AMPK and the endocrine resistance pathway. The common potential health problems linked to these two conditions have been determined (mobility disorders, falls, and death). Deficiencies of certain nutrients determining these conditions, such as dietary deficiencies of energy, protein, and vitamin D_3_, are indicated. It is important to include other etiological factors in the pathophysiology of these diseases, which are still poorly recognized. Such factors include dietary patterns. Our study found an association between the incidence of suspected sarcopenia and frailty syndrome and unfavorable dietary patterns, i.e., consumption of refined cereal products, butter and potatoes, and processed protein products (cheese, cured meats, smoked sausages, and hot dogs). In contrast, a more favorable dietary pattern associated with frequent fruit and water consumption reduced the risk of these conditions. To this end, further research into the mechanisms involved in the development of sarcopenia and frailty syndrome and their mutual coexistence should consider nutritional factors, including dietary patterns. The issue is the need for representative study sampling and prospective cohort studies, including clinical trials. With this approach and further discoveries, it will be possible to establish a common approach to strategies for preventing and treating these two health disorders with dietary management.

## Figures and Tables

**Table 1:** Characteristics of the study sample.

Total		Total	
		N *	%
		172	100.0
Gender	Men Women	36 136	20.9 79.1
Age (years)	65 and below 66–70 71–75 above 75	27 43 44 58	15.7 25.0 25.6 33.7
Education	Primary Vocational Secondary High	37 82 36 17	21.5 47.7 20.9 9.9
Place of residence	A village A town with less than 100,000 inhabitants A city with 100,000 plus inhabitants	17 24 131	9.9 14.0 76.1
Residential status	Care institution I live alone I live with my partner I live with my family without a partner I live with my family and my partner	39 68 42 16 7	22.7 39.5 24.4 9.3 4.1

* N—number of participants.

**Table 2:** Factor loading matrix for the dietary patterns (factors) identified by principal component analysis.

Food Groups	Factors										
	1	2	3	4	5	6	7	8	9	10	11
White bread and bakery products, e.g., wheat bread, rye bread, wheat–rye bread, etc.	0.753 *	0.016	−0.053	−0.058	−0.126	−0.053	0.155	0.099	−0.064	−0.117	0.154
White rice; white pasta; fine-ground groats, e.g., semolina, couscous	0.518 *	0.244	−0.255	−0.206	0.167	0.110	−0.108	−0.084	−0.036	−0.175	−0.089
Butter as a bread spread or as an addition to your meals, for frying, for baking, etc.	0.605 *	−0.025	0.192	0.117	−0.294	0.074	0.011	−0.189	0.146	0.023	−0.048
Potatoes (excluding chips and crisps)	0.550 *	0.264	−0.034	−0.119	0.047	0.032	−0.207	0.086	0.137	0.050	−0.041
Fruit juices	0.064	0.871 *	0.050	0.108	−0.038	−0.042	−0.002	0.081	0.018	0.040	0.045
Vegetable juices	0.060	0.719 *	−0.094	0.097	0.202	−0.032	0.091	−0.152	0.037	0.147	0.028
Sweetened carbonated or drinks such as Coca-Cola, Pepsi, Sprite, Fanta	0.132	0.535 *	0.047	−0.249	0.053	0.270	−0.168	0.029	0.005	−0.297	−0.067
Fermented milk drinks, e.g., yoghurts, kefir (natural or flavored)	−0.134	0.129	0.677 *	0.138	0.234	−0.067	0.119	−0.146	0.015	0.018	−0.017
Fresh cheese curd products, e.g., cottage cheese, cream cheese	0.062	−0.096	0.803 *	−0.091	0.057	−0.029	−0.232	0.054	0.048	−0.033	−0.018
Eggs	−0.049	−0.110	0.055	0.672 *	0.413	−0.081	0.010	−0.043	0.062	−0.084	0.008
Tinned (jar) vegetables, e.g., pickle	−0.048	0.245	−0.081	0.715 *	−0.157	0.068	0.120	0.190	−0.086	0.085	0.055
Fish	−0.023	0.151	0.205	0.035	0.665 *	0.174	0.027	−0.097	−0.168	0.090	−0.033
Legume-based foods, e.g., beans, peas, soybeans, lentils	−0.154	0.061	0.030	0.070	0.715 *	−0.114	0.098	0.045	0.097	0.044	0.005
Red meat, e.g., pork, beef, veal, lamb, game	0.265	−0.193	0.046	0.061	−0.030	0.567 *	−0.080	0.093	0.147	0.334	0.055
Tinned (jar) meats	−0.091	0.022	−0.087	−0.045	−0.028	0.641 *	0.046	0.028	0.445	−0.104	0.035
Energy drinks such as Red Bull, Monster, Rockstar, or other	−0.016	0.096	−0.080	0.005	0.026	0.795 *	0.021	−0.026	−0.127	−0.038	−0.011
Fruit	−0.144	0.110	0.289	0.257	0.079	−0.045	0.521 *	−0.043	−0.068	−0.071	0.406
Water, e.g., mineral water, tap water	0.098	−0.058	−0.086	0.075	0.094	0.075	0.754 *	0.038	−0.084	0.010	−0.164
Lard as a bread spread, or as an addition to your meals, for frying, for baking, etc.	0.009	0.030	0.104	0.091	−0.219	0.193	−0.031	0.666 *	−0.166	−0.183	0.005
Instant soups or ready-made soups, e.g., tinned, jar, concentrates	−0.001	−0.048	−0.139	−0.001	0.152	−0.092	−0.069	0.785 *	0.046	0.019	0.000
Cheese (including processed cheese, blue cheese)	−0.011	0.217	0.117	−0.206	−0.007	−0.077	−0.166	−0.061	0.689 *	0.019	0.149
Cured meat, smoked sausages	0.426	−0.177	0.010	0.193	0.009	0.115	−0.085	−0.037	0.639 *	−0.033	−0.059
Whole wheat (brown) bread/bread roll	−0.080	0.030	0.017	0.018	0.026	0.037	0.021	−0.023	0.053	0.822 *	−0.074
Fast foods, e.g., potato chips/French fries, hamburgers, pizza, hot dogs	−0.001	0.054	−0.130	0.037	−0.074	−0.003	−0.091	0.008	0.086	−0.012	0.825 *
Variance explained (%)	10.5	8.5	7.4	6.1	5.4	4.9	4.5	4.0	3.6	3.6	3.3
Total variance explained (%)	61.8										

* Correlations coefficients higher than 0.5.

**Table 3:** Dietary patterns in the study group.

Factors *	Median	Range
1. White bread and bakery products, white rice and pasta, butter, potatoes	0.66	0–2
2. Fruit and vegetable juices, sweetened carbonated drinks	0.07	0–2
3. Fermented milk drinks, fresh cheese curd products	0.50	0–2
4. Eggs, tinned vegetables	0.25	0–2
5. Fish, legume-based foods	0.10	0–2
6. Red meat, tinned meats, energy drinks	0.04	0–2
7. Fruit, water	1.50	0–2
8. Lard, instant soups	0.00	0–2
9. Cheese, cured meat, smoked sausages	0.21	0–2
10. Whole wheat bread	0.14	0–2
11. Fast foods	0.00	0–2

* Identified by factor analysis on the basis of daily frequency of consumption: 0.00—never, 0.06—1–3 times a month, 0.14—once a week, 0.5—a few times a week, 1—once a day, 2—a few times a day.

**Table 4:** Characteristics of the study group according to sarcopenia and frailty syndrome.

	Total Sample % (N) *	Gender		Age			
		Women, % (N)	Men, % (N)	65 and below, % (N)	66–70, % (N)	71–75, % (N)	Over 75, % (N)
SARC-F ** no (<4 points) yes (≥4 points)		<0.001 ***		0.004			
	68.0 (117)	76.5 (104)	36.1 (13)	59.3 (16)	83.7 (36)	77.3 (34)	53.4 (31)
	32.0 (55)	23.5 (32)	63.9 (23)	40.7 (11)	16.3 (7)	22.7 (10)	46.6 (58)
GST ≤0.8 m/s >0.8 m/s		<0.001		0.007			
	69.2 (119)	75.7 (103)	44.4 (16)	63.0 (17)	86.0 (37)	75.0 (33)	55.2 (32)
	30.8 (53)	24.3 (33)	55.6 (20)	37.0 (10)	14.0 (6)	25.0 (11)	44.8 (26)
HGS ≥16 kg (W) * ≥27 kg (M) * <16 kg (W) * <27 kg (M) *		<0.001		0.213			
	83.7 (144)	89.7 (122)	61.1 (22)	85.2 (23)	93.0 (40)	81.8 (36)	77.6 (45)
	16.3 (28)	10.3 (14)	38.9 (14)	14.8 (4)	7.0 (3)	18.2 (8)	22.4 (13)
FTSST ≤15 s >15 s		0.001		0.020			
	69.2 (119)	75.0 (102)	47.2 (17)	70.4 (19)	83.7 (36)	72.7 (32)	55.2 (32)
	30.8 (53)	25.0 (34)	52.8 (19)	29.6 (27)	16.3 (7)	27.3 (12)	44.8 (26)
EFS non-frail pre-frail or frail		0.015		<0.001			
	54.1 (93)	58.8 (80)	36.1 (13)	63.0 (17)	76.7 (33)	50.0 (22)	36.2 (21)
	45.9 (79)	41.2 (56)	63.9 (23)	37.0 (10)	23.3 (10)	50.0 (22)	63.8 (37)

* N—number of participants; ** SARC-F—sarcopenia risk assessment questionnaire: strength (S), assistance in walking (A), rising from a chair (R), climbing stairs (C), falls (F); GST—gait speed test; HGS—hand grip strength; FTSST—five times sit-to-stand test; EFS—Edmonton Frail Scale; *** *p*-value according to chi-square test.

**Table 5:** Odds ratios for the SARC-F, GST, FTSST, HGS, and EFS according to gender, age, and dietary patterns (factors) in the study sample.

Variables	β *	Exp(β) *	95% CI *		*p*-Value **
Model 1. SARC-F (ref. < 4 points)					
Gender (ref. women)	1.63	5.10	1.95	13.33	<0.001
Age (≤65)	0.07	1.07	1.01	1.13	0.021
Model 2. GST (ref. *n* > 0.8 m/s)					
Factor 1. White bread and bakery products, white rice and pasta, butter, potatoes (ref. non-factor 1)	4.71	111.49	8.18	1519.91	<0.001
Factor 7. Fruit, water (ref. non-factor 7)	−1.65	0.19	0.06	0.60	0.004
Factor 9. Cheese, cured meat, smoked sausages (ref. non-factor 9)	5.69	296.26	12.64	6943.85	<0.001
Gender (ref. women)	1.18	3.25	1.15	9.22	0.027
Model 3. FTSST (ref. ≤ 15 s)					
Gender (ref. women)	1.12	3.06	1.26	7.44	0.013
Age (ref. ≤ 65)	0.06	1.06	1.01	1.12	0.020
Model 4. HGS (ref. ≥ 16 kg (W) and ≥27 kg (M))					
Gender (ref. women)	2.21	9.13	2.92	28.51	<0.001
Model 5. EFS (ref. ≤ 4 points)					
Factor 1. White bread and bakery products, white rice and pasta, butter, potatoes (ref. non-factor 1)	2.43	11.37	1.20	107.52	0.034
Factor 7. Fruit, water (ref. non-factor 7)	−1.65	0.19	0.07	0.52	0.001
Age (ref. ≤ 65)	0.08	1.08	1.03	1.15	0.004

* OR—point estimate (β e^β^), 95% confidence interval; ** significance level according to Wald’s test; W—woman, M—man.

**Table 6:** Odds ratios for the cluster with SARC-F, gender, dietary patterns (factors), and EFS in the study sample.

Variables	Model 6. SC (ref. nSC)				
	β *	Exp(β) *	95% CI *		*p*-Value **
EFS (ref. ≤ 4 points)	2.80	16.45	5.46	49.59	<0.001
Gender (ref. women)	1.77	5.86	1.89	18.17	0.002
Factor 1. White bread and bakery products, white rice and pasta, butter, potatoes (ref. non-factor 1)	2.55	12.82	0.99	165.80	0.050
Factor 9. Cheese, cured meat, smoked sausages (non-factor 9)	3.60	36.63	1.45	924.83	0.029

* OR—point estimate (β eβ), 95% confidence interval; ** significance level according to Wald’s test.

## Data Availability

The data presented in this study are available on request from the corresponding author.

## Appendix A

**Table A1:** Odds ratios for the SARC-F according to gender, age, and all analyzed dietary patterns (factors) in the study sample.

Variables	β *	Exp(β) *	95% CI *		*p*-Value **
Model 1. SARC-F (ref. < 4 points)					
Factor 1	0.74	2.10	0.70	6.29	0.185
Factor 2	0.76	2.14	0.66	6.96	0.207
Factor 3	−1.30	0.27	0.07	1.06	0.060
Factor 4	−1.11	0.33	0.04	2.49	0.282
Factor 5	−0.65	0.52	0.02	11.73	0.683
Factor 6	1.46	4.30	0.12	148.50	0.420
Factor 7	−1.11	0.33	0.05	0.73	0.086
Factor 8	−0.83	0.44	0.04	4.59	0.490
Factor 9	0.85	2.34	0.27	19.9891	0.439
Factor 10	0.65	1.91	0.97	3.77	0.061
Factor 11	−3.78	0.02	0.00	334.2870	0.440
Gender (ref. women)	1.63	5.10	1.95	13.33	<0.001
Age	0.07	1.07	1.01	1.13	0.021
Constant	−6.01	0.01			0.004

* OR—point estimate (β e^β^), 95% confidence interval; ** significance level according to Wald’s test.

**Table A2:** Odds ratios for the GST according to gender, age, and all analyzed dietary patterns (factors) in the study sample.

Variables	β *	Exp(β) *	95% CI *		*p*-Value **
Model 2. GST (ref. *n* > 0.8 m/s)					
Factor 1	4.71	111.49	8.18	1519.91	<0.001
Factor 2	0.28	1.32	0.35	4.93	0.681
Factor 3	−0.89	0.42	0.11	1.67	0.221
Factor 4	−1.68	0.19	0.02	2.06	0.170
Factor 5	0.42	1.52	0.06	35.91	0.796
Factor 6	−3.61	0.03	0.001	1.43	0.075
Factor 7	−1.65	0.19	0.06	0.60	0.004
Factor 8	0.27	1.31	0.16	10.43	0.798
Factor 9	5.69	296.26	12.64	6943.85	<0.001
Factor 10	0.86	2.35	1.19	4.87	0.081
Factor 11	−12.29	0.00	0.00	6977.69	0.254
Gender (ref. women)	1.18	3.25	1.15	9.22	0.027
Age	0.03	1.02	0.99	1.08	0.403
Constant	−3.65	0.03			0.104

* OR—point estimate (β e^β^), 95% confidence interval; ** significance level according to Wald’s test.

**Table A3:** Odds ratios for the FTSST according to gender, age, and all analyzed dietary patterns (factors) in the study sample.

Variables	β *	Exp(β) *	95% CI *		*p*-Value **
Model 3. FTSST (ref. ≤ 15 s)					
Factor 1	0.80	2.23	0.79	6.30	0.128
Factor 2	0.07	1.07	0.34	3.36	0.910
Factor 3	−1.14	0.32	0.08	1.21	0.094
Factor 4	0.20	1.22	0.20	7.32	0.826
Factor 5	−2.33	0.10	0.01	2.45	0.157
Factor 6	−0.06	0.94	0.03	25.44	0.973
Factor 7	−0.251	0.78	0.37	1.63	0.506
Factor 8	−0.085	0.92	0.11	7.65	0.937
Factor 9	1.813	6.13	0.77	48.67	0.086
Factor 10	0.231	1.26	0.67	2.38	0.476
Factor 11	−13.154	0.00	0.00	199.61	0.162
Gender (ref. women)	1.12	3.06	1.26	7.44	0.013
Age (ref. ≤ 65)	0.06	1.06	1.01	1.12	0.020
Constant	−6.690	0.001			<0.001

* OR—point estimate (β e^β^), 95% confidence interval; ** significance level according to Wald’s test.

**Table A4:** Odds ratios for the HGS according to gender, age, and all analyzed dietary patterns (factors) in the study sample.

Variables	β *	Exp(β) *	95% CI *		*p*-Value **
Model 4. HGS (ref. ≥ 16 kg (W) and ≥ 27 kg (M))					
Factor 1	0.53	1.70	0.43	6.74	0.450
Factor 2	−0.24	0.78	0.14	4.43	0.784
Factor 3	0.34	1.40	0.32	6.07	0.650
Factor 4	−1.88	0.15	0.01	3.66	0.247
Factor 5	1.96	7.14	0.24	214.19	0.257
Factor 6	0.06	1.06	0.02	53.17	0.977
Factor 7	−0.54	0.58	0.22	1.51	0.266
Factor 8	−1.57	0.21	0.01	19.07	0.496
Factor 9	2.06	7.88	0.66	94.42	0.103
Factor 10	−1.29	0.27	0.09	0.85	0.025
Factor 11	−8.20	0.00	0.00	1793.09	0.391
Gender (ref. women)	2.21	9.13	2.92	28.51	<0.001
Age	0.05	1.05	0.98	1.12	0.147
Constant	−7.66	0.00			0.004

* OR—point estimate (β e^β^), 95% confidence interval; ** significance level according to Wald’s test; W—woman, M—man.

**Table A5:** Odds ratios for the EFS according to gender, age, and all analyzed dietary patterns (factors) in the study sample.

Variables	β *	Exp(β) *	95% CI *		*p*-Value **
Model 5. EFS (ref. ≤ 4 points)					
Factor 1	2.43	11.37	1.20	107.52	0.034
Factor 2	0.25	1.28	0.39	4.24	0.680
Factor 3	−1.15	0.32	0.10	1.02	0.055
Factor 4	−0.52	0.59	0.10	3.57	0.567
Factor 5	1.37	3.95	0.28	55.14	0.307
Factor 6	0.46	1.59	0.05	50.18	0.793
Factor 7	−1.65	0.19	0.07	0.52	0.001
Factor 8	−0.09	0.91	0.12	6.98	0.929
Factor 9	0.78	2.19	0.27	17.78	0.462
Factor 10	0.67	1.95	0.98	3.86	0.055
Factor 11	−16.46	0.00	0.00	8.62	0.083
Gender (ref. women)	0.44	1.55	0.61	3.94	0.357
Age	0.08	1.08	1.03	1.15	0.004
Constant	−6.22	0.00			0.003

* OR—point estimate (β e^β^), 95% confidence interval; ** significance level according to Wald’s test.
